# Microbiological profiles of sputum and gastric juice aspirates in Cystic Fibrosis patients

**DOI:** 10.1038/srep26985

**Published:** 2016-06-01

**Authors:** H. Al-momani, A. Perry, C. J. Stewart, R. Jones, A. Krishnan, A. G. Robertson, S. Bourke, S. Doe, S. P. Cummings, A. Anderson, T. Forrest, S. M. Griffin, M. Brodlie, J. Pearson, C. Ward

**Affiliations:** 1 Institutes of Cellular Medicine and Cell & Molecular Biosciences, Newcastle University Medical School, Newcastle University, Newcastle upon Tyne. NE2 4HH, UK; 2 Department of Microbiology, Newcastle upon Tyne Hospitals NHS Foundation Trust, Freeman Hospital, Newcastle upon Tyne NE7 7DN, UK; 3 Northumbria University, Ellison Place, Newcastle-upon-Tyne NE1 8ST, UK; 4 Adult Cystic Fibrosis Centre and Northern Oesophago-Gastric Unit, Royal Victoria Infirmary, Newcastle upon Tyne NE1 4LP, UK; 5 School of Science and Engineering, Teesside University, Middlesbrough, TS1 3BA, UK

## Abstract

Gastro-Oesophageal Reflux (GOR) is a key problem in Cystic Fibrosis (CF), but the relationship between lung and gastric microbiomes is not well understood. We hypothesised that CF gastric and lung microbiomes are related. Gastric and sputum cultures were obtained from fifteen CF patients receiving percutaneous endoscopic gastrostomy feeding. Non-CF gastric juice data was obtained through endoscopy from 14 patients without lung disease. Bacterial and fungal isolates were identified by culture. Molecular bacterial profiling used next generation sequencing (NGS) of the 16S rRNA gene. Cultures grew bacteria and/or fungi in all CF gastric juice and sputa and in 9/14 non-CF gastric juices. *Pseudomonas aeruginosa(Pa)* was present in CF sputum in 11 patients, 4 had identical *Pa* strains in the stomach. NGS data from non-CF gastric juice samples were significantly more diverse compared to CF samples. NGS showed CF gastric juice had markedly lower abundance of normal gut bacteria; *Bacteroides* and *Faecalibacterium,* but increased *Pseudomonas* compared with non-CF. Multivariate partial least squares discriminant analysis demonstrated similar bacterial profiles of CF sputum and gastric juice samples, which were distinct from non-CF gastric juice. We provide novel evidence suggesting the existence of an aerodigestive microbiome in CF, which may have clinical relevance.

Cystic Fibrosis (CF) is the most common recessively inherited condition in the Caucasian population^1^. Seminal reports from the 1930s described pancreatic abnormalities and steatorrhea, with the disease initially known as “Cystic fibrosis of the pancreas”^2^.

In the modern era CF is recognised as a multi-system disorder. Most of the morbidity and premature mortality associated with CF can be attributed to chronic lung diseases caused by microbial infections and subsequent inflammation^3^, most frequently by *Pseudomonas aeruginosa* (*Pa*)^4^. Treatment from dedicated CF centres has been associated with increased survival and treatment of lung disease is an understandable priority. The overall benefits associated with dedicated CF centres however emphasise a multi-disciplinary approach, and gastrointestinal problems are recognised as prominent. In particular Gastro-Oesophageal Reflux (GOR) is increased with a prevalence of more than 50%^5^. Several mechanisms have been proposed including reduced lower oesophageal sphincter pressure (LOS), increased transient LOS relaxation, and delayed gastric emptying. Increased GOR might also be due to an increased abdomino-thoracic pressure gradient during cough and physiotherapy^6^.

A relationship between aspiration of gastric contents during a reflux event and deterioration of lung function is implied by the finding of poorer lung function in CF patients with acid reflux^7^. Anti-reflux medications have been associated with improved respiratory symptoms and lung function and anti-reflux surgery with a slower lung function decline and significant decrease in CF exacerbations^8^. However it has also been suggested in a scarce literature that treatment with the proton pump inhibitor esomeprazole (PPI) may be associated with earlier and more frequent exacerbations compared with placebo^9^.

The potential importance of an aero-digestive microbiome has long been the subject of interest in respiratory and gastrointestinal medicine in situations other than CF. Multiple reports provide evidence that a gastric reservoir is a risk factor for acquiring nosocomial pneumonia in the intensive care unit (ICU) setting^10^. Studies have also been carried out in elderly, non-CF patients which report both concordance between gut and respiratory bacteria and a high prevalence of bacteria present in the gut prior to their presence in the respiratory tract^11^. This data is consistent with a link between gastric and lower respiratory tract bacterial colonisation in ICU patients fed by nasogastric tube^12^.

The microbial colonisation of stomach contents in CF patients and its potential to act as a reservoir for microorganisms known to cause lung infection is not completely understood. We therefore undertook a study of the microbiology of CF lung and gastric contents. Our hypothesis was that bacterial flora in sputum would be related to that in gastric aspirate.

## Methods

### Ethical approval

This study was adopted by the Hepatopancreatobiliary and Gastroenterology Biobank, Newcastle University and approved by the Newcastle & North Tyneside 1 Research Ethics Committee (UK). All study participants provided written informed consent prior to initiation of the study. All methods were carried out in accordance with relevant guidelines.

### Symptoms of extraoesophageal reflux

Patients with CF had symptoms of extraoesophageal reflux (EOR) assessed using the Reflux Symptoms Index (RSI) score; score less than 13 classed as not EOR symptomatic^13^.

### CF gastric juice and sputum samples

CF patients receiving percutaneous endoscopic gastrostomy (PEG) feeding represented an important opportunity to directly sample gastric juice, with less potential for contamination with oropharnygeal commensals. Only adult stable CF patients receiving PEG tube feeding, from the 270 CF patients attending the regional CF Centre, were therefore included in this study; potentially 18 patients from the regional clinic population. Following overnight fasting, gastric juice was collected from 15 sequential patients who attended the clinic during the course of the study between 22/05/2013-17-04/2014, 83% of all the potentially available patients with PEG tubes in the region were therefore included in this study. No PEG fed CF patients were excluded or positively selected for the study (Table 1).

In brief, 10 ml of sterile saline was injected through the PEG and aspirated after 2-3 minutes. Spontaneously expectorated sputum (n = 13) and cough swabs (n = 2) were also obtained.

Gastric juice pH was measured using pH strips and sputum samples were homogenised with equal amounts of dithiothreitol for 1 to 3 minutes.

### Collection of non-CF gastric juice samples

Following fasting (≥8 hours) gastric juice was collected from 14 patients without CF undergoing routine upper gastro-intestinal endoscopy performed according to British Society of Gastroenterology guidelines, by suctioning gastric juice through the endoscope (Table 2). All patients were requested to stop any acid suppression medication 2 weeks before the endoscopy procedures. Sputum was not collected from the non-CF patients.

### Gastric juice and sputum samples microbial study

Gastric juice and sputum samples underwent microbiological culture performed in accordance with UK standard methods. An aliquot was stored at −20 °C for DNA extraction.

10 μL of homogenised sputum and gastric juice were plated for bacterial and fungal cultures. The following media were used: Columbia blood agar supplemented with 5% horse blood, chocolate agar supplemented with 70 mg/L bacitracin, Burkholderia selective agar (for CF sputa and gastric juice, CEP bioMérieux UK), Cysteine lactose electrolyte deficient agar (CLED) and fastidious anaerobic agar (FAA). Plates were incubated according to standard protocol, CEP cultures were incubated for 10 days at 30 °C for isolation of *Burkholderia cepacia* complex and rapidly growing mycobacterium.

Plates were examined daily for evidence of microbial growth and assessment of the number of distinct colonial variants was recorded. All morphological variants were sub-cultured and used for identification and stored at −20 °C in 10% glycerol skimmed milk. Isolates were identified by matrix assisted laser desorption ionisation time-of-flight (MALDI-TOF) mass spectrometry (Bruker Daltonics, UK) and where necessary, appropriate API kits (bioMérieux, UK)^14^. Mycobacterium was identified by *rpo*B, *sodA* and *hsp*65 gene sequencing and strain typed using variable number tandem repeat (VNTR), (Colindale, UK^15^). All isolates of *Pa* were typed via VNTR profiling^16^.

### DNA extraction

DNA extraction was performed from sputum and gastric juice samples using a PowerSoil™ DNA Isolation Kit (MoBio) in accordance with the manufacturer’s instructions.

### Molecular based studies

Bacterial profiling utilised the 16S rRNA gene targeting variable region 4 (V4) based on the Schloss wet-lab MiSeq SOP (http://www.mothur.org/wiki/MiSeq_SOP). Raw fastq data were processed using Mothur (version 1.31.2) as described in the MiSeq SOP^17^. Chimeric sequences were detected by Chimera.uchime and removed from downstream analysis. Alignment was generated via the Silva database^18^. A cutoff of 70 was applied to assign sequences to the trainset_ 9_032012 resulting in 2,228,291 reads. All sequences were deposited in MG-RAST under the accession numbers 4603845.3 - 4603893.3.

### Statistical analysis

NGS profiles were analysed by multivariate partial least squares discriminant analysis (PLS-DA) (SIMCA 13.0 software, Stockholm, Sweden)^19^. PLS-DA uses assigned variables to interrogate data for maximum variance. To check data was adhering to multivariate normalities, Hotelling’s T2 tolerance limits were calculated and set at 0.95.

## Results

### CF Patients

We sampled over 80% of all the PEG patients potentially available in the North East Region of England. This patient cohort had severity of CF lung disease in keeping with this population (median FEV1, 1.55 L range 0.45–3.5 L) along with long term antibiotic exposure and use of acid suppression medication for all patients (Table 1).

### Microbial culture

Routine microbial culture was positive for bacteria and/or fungi in all CF gastric juice and sputum samples. All samples had more than one organism isolated except a gastric juice sample from CF-8 that only had *Candida albicans*. See table E1 and E2 in the online data supplement for CF gastric juice and CF sputum samples culture results.

### Symptoms of extraoesophageal reflux in CF patients

Symptom scores for extraoesophageal reflux were available in 12 of the 15 patients with CF (Table 1). All patients were EOR symptomatic, with an RSI score >12; median RSI score 17 (range 13–36).

### CF gastric juice vs sputum samples culture results

Nine of the 15 CF patients included in this study had one or more microorganism (fungal or bacteria) common between sputum and gastric juice samples, 5 had at least one bacteria taxa common between sputum and gastric juice while 4 had one or more fungal species. Among the bacterial common between sputum and gastric juice were, *Pa* (4/9), *Streptococcus* spp (2/9) and *Achromobacter* spp. (2/9). Conversely, 6 patients had microorganisms isolated in both gastric juice and sputum, with no organism common to both samples.

All CF patients had fungal species isolated in either sputum or gastric juice samples, this included 8 patients with *Candida* spp. One patient had *Aspergillus* spp. isolated in both gastric and sputum samples.

### CF gastric juice vs non-CF gastric juice culture results

Microorganisms, either bacterial or fungal, were isolated from all 15 CF gastric juice samples. No microorganisms were isolated from 5 of the 14 non-CF gastric juice samples. (see Table E3).

Bacterial species were detected in 11/15 CF gastric juice samples (mean 2.2 bacterial isolates/patient). *Streptococcus* spp. (4/15), *Pa* (4/15), *Lactobacillus* spp. (4/15), and *Staphylococcus* spp. (3/15) were the most frequent bacteria isolated. Bacterial species were detected in 8/14 non-CF gastric juice samples (mean 2.2 bacteria isolates/patient). *Streptococcus* spp (4/14), *Lactobacillus* spp (2/14) S*taphylococcus* spp (2/14) were the most frequently isolated. *Pa* was detected in only one patient, without CF or other underlying lung disease. Fungal pathogens were detected in all CF gastric juice samples (mean of 2 fungi /patient), *Candida* spp were isolated from only 3 out of 14 non-CF gastric juice samples.

#### Pseudomonas aeruginosa

*Pa* was detected in 11 of the 15 CF sputa (73%) (CF-1, CF-5, CF-6 and CF8-15) and in 4 of the 15 CF gastric juice samples (26%) (CF-1, CF-5, CF-12 and CF-15). CF-1 and CF-12 had both mucoidal and non-mucoidal *Pa* in both sputum and the gastric juice, CF-5 had mucoidal and non-mucoidal *Pa* in sputum and only mucoidal *Pa* in gastric juice, CF-15 had only mucoidal *Pa* in sputum and gastric juice.

The median age of patients with and without Pa was 26 (range 20–27) and 23 (range 18–41) respectively. The median FEV1 for patients with *Pa* was 1.55L (range 0.45–3.5L) compared to 1.25L (range 0.8–2.7L) in non *Pa* infected patients.

Variable number tandem repeat (VNTR) analysis of *Pa* showed that *Pa* strains isolated from gastric juice and sputum samples were identical in 3 of the 4 patients who cultured *Pa* in both gastric juice and sputum. The remaining CF patient with *Pa* in gastric juice and sputum (CF-1) also appeared to have matched strains of *Pa* in both sputum and gastric juice. However, in addition, this patient also had other strains of *Pa* present only in sputum samples and not isolated in gastric juice.

### Next Generation Sequencing

**Figure 1:**
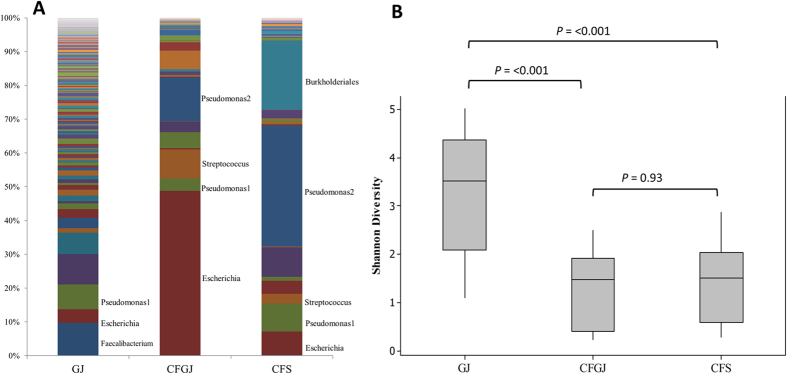
(**A**) Bar plot showing the relative abundance of each operational taxonomic unit (OTU) within non-CF gastric juice (GJ), CF gastric juice (CFGJ), and sputum samples (CFS). (**B**) Shannon Diversity Index of CF samples (CFGJ and CFS) and non-CF GJ.

Fourteen non-CF gastric juice (GJ 1-14), 13 CF gastric juice (CF-GJ 1, 2, 4-12, 14, 15) and 12 CF sputum samples (CFS 4-15) had microbiome analysis using NGS. Non-CF gastric juice showed higher diversity compared to the CF gastric juices and sputum samples (Fig. 1A). Proteobacteria were abundant in both CF gastric juice and CF sputum samples (72% and 74% of relative abundance, respectively) whereas Firmicutes were more abundant in non-CF gastric juice (48%).

The average Shannon diversity indices (*H’*) was significantly higher in non-CF gastric juice than CF gastric juices (*P* = 0.002) and CF sputum (*P* = < 0.001). The *H’* of CF gastric juice and sputum samples were not different (P = 0.93: Fig. 1B).

**Figure 2:**
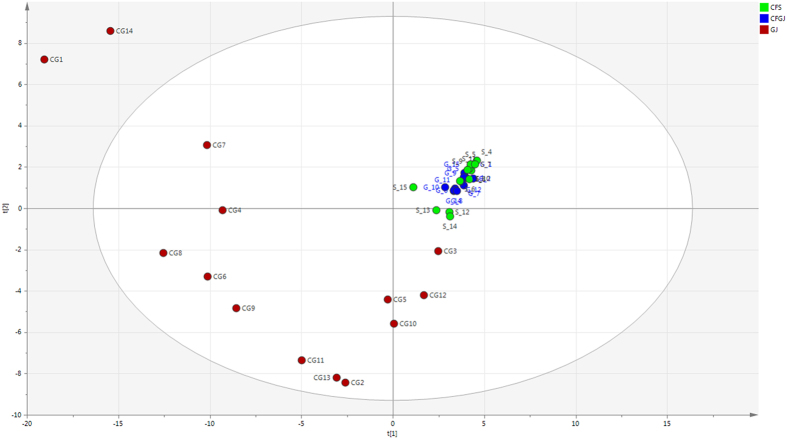
Partial least square discriminant analysis (PLS-DA) score scatter plot of all samples. Non-CF gastric juice – GJ; CF gastric juice – CFGJ; Sputum samples – CFS. The Axes represent a % of variance. The CF gastric juice and sputum samples show a close relationship. In contrast the Gastric Juice samples from control patients are widely variable and clearly distinct from the CF sputum and gastric juice clustering.

PLS-DA of all samples demonstrated that both gastric juice and sputum samples from patients with CF had comparable profiles, which was distinct from non-CF gastric juice (Fig. 2). CF samples showed a relative low average of Shannon diversity making them cluster near the origin of PLS-DA plots, while non-CF gastric juice samples showed high diversity. Further analysis of the matched gastric juice and sputum samples from the same patient showed these samples generally grouped together (see Figure E1 in the data supplement). The loading plot from the PLS-DA of the overall bacterial community revealed that *Prevotella, Rothia, Streptococcus, Staphylococcus, Herminiimonas* and *Pseudomonas* were closely associated with CF samples while *Faecalibacterium, Roseburia, Bacteroides,* and *Lactobacillus* were more associated with non-CF gastric juices (see Figure E2 in the data supplement).

## Discussion

We believe this is the first study directly comparing microorganisms isolated from the airways and gastric juice in the same PEG fed CF patients. We showed that the digestive tract and airways were populated by bacteria which were sometimes identical and of known importance in CF lung disease, including biofilm forming strains of *Pa*. This highlights the possibility that the stomach constitutes a viable bacterial reservoir, relevant to the overall pathophysiology of CF^20^.

We found that gastric juice samples from adult CF patients were significantly different from non-CF gastric juice samples. At a molecular level, NGS of CF gastric juice showed a much lower abundance of bacteria than typically found in the normal stomach, with CF gastric juice containing *Bacteroides*, *Faecalibacterium* and increased amounts of *Pa* compared with the non-CF controls^21^. The results obtained from our non-CF gastric juice samples were in accordance with the known make-up of the normal gastric microbiota^22^.

In all cases where *Pa* was found concordantly in CF gastric juice and sputum we demonstrated genetically identical strains by VNTR. If lung and stomach were colonised by *Pa* from unrelated, stochastic sources, the organisms would be highly unlikely to be identical at the molecular level. In CF patients with intermittent colonization or recently acquired chronic *Pa* infection, there are high levels of genotype diversity^23^, suggesting that CF patients acquire unique *Pa* strains independently, from different environmental sources^24^. For intermittently colonized patients, following initial eradication by inhaled antibiotic treatment, recolonization by *Pa* can occur. This can be with a different genotype, suggesting new environmental sources, but in approximately 25% of patients the same genotype is identified, suggesting either undetectable persistent colonisation or local unknown environmental sources of recolonization^23^. Careful longitudinal eradication studies in CF have observed patients being re-colonized with the original *Pa* strains after several years of no demonstrable *Pa* in sputa^25^. The findings reported in this study might be consistent with the notion that gastric contents may represent a niche, relatively protected from inhaled antibiotics used in best practice eradication protocols^26^.

Our PEG tube gastric juice sampling eliminated the possibility of contamination by upper gastro intestinal tract and respiratory flora. These are potential considerations for other sampling procedures, such as endoscopy. Our data unambiguously confirms the ability of bacteria to survive in the stomach, constituting a potential reservoir of viable pathogens relevant to CF disease. Reflux and aspiration events have been widely documented in CF patients^27^, and our CF patients had symptoms of extraoesophageal reflux beyond the normal range. Our data might therefore be consistent with the possibility that microorganisms relevant to CF lung pathophysiology could be aspirated.

It is also likely that gastric juice *Pa* may derive from swallowed sputum containing *Pa* that is cleared from the lung following cough. We found one patient where identical *Pa* was found in sputum and gastric juice but where the sputum sample also contained *Pa* not isolated in gastric juice. Alongside our finding of *Pa* in the gastric juice of a control subject without lung disease, our data indicate that although Gastric juice *Pa* may be swallowed following mucocilliary clearance of a diseased airway, *Pa* could be derived through diverse routes and sources, including but not restricted to microaspiration for lung *Pa*.

It has long been recognised that as well as refluxing, even healthy adults may aspirate oropharyngeal secretions, especially during reclined sleep. An indium 111 chloride technique from 1978 showed that 45% of normal subjects aspirated during deep sleep, and that in patients with “depressed consciousness”, aspiration was detectable in 70% of patients^28^. This study noted the potential for bacterial pneumonia as a result of failed clearance of aspirated bacteria. A further valuable quantitative study by Gleeson and colleagues employed infusion of 2 mL/h radioactive Tc-99m tracer into the nasopharynx to estimate the quantity of occult aspiration of nasopharyngeal secretions in normal humans. Aspiration was common, occurring in 50% of healthy young men during sleep, and was variable within subjects studied on more than one occasion. The levels of aspiration measured were 0.01 to 0.2 mL; levels noted as being consistent with a potentially significant bacterial inoculum^29^.

Microbiological continuity of the aerodigestive tract, using culture independent methods has also previously been suggested in healthy adults indicating that microaspiration may be common in healthy individuals^30^. An association between GOR and lower respiratory tract *Pa* colonisation in children with CF is also consistent with potential gastric immigration^31^. More recently in children with chronic cough undergoing bronchoscopy and gastrointestinal endoscopy 8 of the most abundant bacteria in the gastric fluid were shown to be abundant in the lungs. The authors concluded that this represented evidence of microbiological exchange between the lung and the gastrointestinal tract, independent of the oropharyngeal microbiome^32^.

It is of interest that molecular epidemiology studies in CF lung transplant recipients show that lung allografts become recolonized with the same clone of *Pa* cultured before transplantation^33^. This recolonization is believed to originate from the upper airway and the sinuses^34^. It is also possible that the lungs of CF patients might be vulnerable to infection from aspiration of *Pa* colonising the gastrointestinal tract post transplantation however^35^, and that bi-directional transmission of pathogenic organisms, including *Pa*, occurs between the stomach and the oropharynx^12^. In lung transplantation, GOR has been substantially implicated as a potential non-alloimmune cause of Bronchiolitis Obliterans Syndrome (BOS), and anti-reflux fundoplication surgery has been assessed^36^, and associated with improved allograft function^37^.

Molecular methods have previously demonstrated that gastric microbiome diversity is normally high and similar to that described for the lower gastrointestinal tract^22^,^38^. Decreasing airway bacterial diversity in patients with CF is well described^39^ . This is potentially consistent with our study showing that the CF gastric microbiota showed lower diversity compared to non-CF in the molecular based approach. This loss of diversity was a common feature between sputum and CF gastric juice samples compared to non-CF gastric juices. Molecular identification and multivariate discriminant analysis showed that the CF gastric juice samples and sputum samples were clustered and distinct from the non-CF gastric juices. Moreover, sputum and CF gastric juice samples from the same patient also had comparable profiles. Overall this further suggests that a link may be present between microbes colonising the sputum and gastric juice in some CF patients and that this is worthy of further investigation. Such studies should investigate whether the same profile of micro-organisms in the sputum and the gastric juice we observed might be explained by a common origin in the sinus and/or oro-pharynx.

There are limitations to our study inherent to a real world clinical setting and our study design. We restricted our gastric juice sampling to PEG fed patients and sampled over 80% of all the PEG patients potentially available to us, but this limited our study and our reported data to a specific patient cohort. There was no exclusion based on severity of the disease or antibiotic exposure. It is relevant to consider that apart from PEG fed patients with CF we have previously isolated *Pa* from samples of gastric juice from non- PEG fed CF patients, where gastric juice was sampled by endoscopy^35^. Our previous study found strains of *Pa* identical at the molecular level in broncho alveolar lavage (BAL), endoscopically sampled gastric juice and sputa. This suggests that the findings of our present study, that the stomach constitutes a viable bacterial reservoir relevant to the overall pathophysiology of CF, is not restricted solely to PEG fed patients^35^.

We also recognise that long term antibiotic exposure and use of Proton Pump Inhibitors (PPIs) in our patients could impact on the gastric juice microbiology that we observed. Use of PPIs is increasingly recognised to be associated with alterations in the gastric, lung, and oropharyngeal Microflora^32^. The importance of this is a source of current debate however, to which we have contributed^40^.

Gastric acid (GA) inhibition via proton pump inhibitors or histamine-2 receptor antagonists is prescribed to CF patients when fat absorption remains insufficient despite an adequate dosage of pancreatic enzyme replacement^41^. Gastroesophageal reflux disease is another reason to start drugs for GA inhibition in patients with CF, and in North America it has been observed that the majority of patients with CF are on gastric acid suppressive treatments^42^. According to the clinical practice in our centre of study and that of others, all our CF patients were therefore on acid suppression therapy. Antibiotic therapy is also a current mainstay of therapy in CF. We feel that further studies regarding the effect of antibiotics and anti-acid therapy on the aerodigestive microbiome in CF, though challenging, could be important. We hope that such work may be aided and informed by our present study data.

In summary, we have shown a novel association between the sputum and gastric juice microbiome of CF patients. This study demonstrates that the stomach may represent an under recognised foci of bacterial infection, and potential reservoir of *Pa* in CF. Gastric microbiology could be contributed to by cough, expectoration and swallowing of organisms from the lung. Patients with CF are also known to reflux and aspirate however, indicating that in principle gastric microorganisms could contribute to the lung compartment. We conclude that the ‘aerodigestive microbiome’ may have potential relevance in the pathophysiology of CF and may have therapeutic implications, since *Pa* eradication does not consider a gastric niche of organisms.

## Additional Information

**How to cite this article**: Al-momani, H. *et al.* Microbiological profiles of sputum and gastric juice aspirates in Cystic Fibrosis patients. *Sci. Rep.* **6**, 26985; doi: 10.1038/srep26985 (2016).

## Supplementary Material

Supplementary Information

## Figures and Tables

**Table 1:** Demographic data for CF patients.

Patient	Genetics	Age	RSI score	PPI yes/no	Gastric Juice pH	FEV1 (% pred)	BMI	IV days /year	Long-term antibiotic
CF-1	F508del/F508del	26	17	Yes	6	2.0L (52%)	19.9	22	Azith Inh Coli Inh Tob
CF-2	F508del/F508del	27	20	Yes	2	1.7 (42%)	23.2	70	Azith Fluclox Inh coli
CF-3	F508del/F508del	20	25	Ranitidine	3	0.8L (26%)	19.5	28	Azith Fluclox Inh Coli
CF-4	F508del/F508del	24	36	Yes	6	0.76L (28%)	19	154	Azith Inh Coli
CF-5	F508del/F508del	41	NA [died]	Yes	5.5	0.45L (18%)	18.2	65	Azith Inh Tob
CF-6	F508del/F508del	31	16	Yes	6	0.5L (12%)	19.1	70	Azith Inh Coli
CF-7	F508del/ R117H	22	16	Yes	3	2.7L (66%)	16.4	14	Fluclox
CF-8	I507del/ Arg560Lys	18	13	Yes	2	3.5L (88%)	19.4	37	Fluclox Inh Coli Inh Tob
CF-9	F508del/R117H	30	14	Yes	6	1.55L (46%)	17.8	56	Fluclox Inh Coli
CF-10	F508del/F508del	25	17	Yes	2	1.7L (38%)	15.9	98	Azith Fluclox Inh Tob
CF-11	F508del/G542X	32	NA [PEG removed]	No	2	1.15L (36%)	19.4	112	Azith Inh Coli
CF-12	F508del/F508del	30	19	Yes	6	1.2L (29%)	19.8	115	Azith Fluclox Inh Coli
CF-13	F508del/G542X	24	15	Yes	2	1.65L (36%)	15.24	197	Azith Inh Coli Inh Tob
CF-14	F508del/ Arg851Ter	23	22	Yes	6	2.3L (59%)	20.2	56	Azith
CF-15	G542X/G551D	22	NA [moved country]	Yes	2	0.85L (28%)	18	42	Azith Inh Coli

Azith = oral azithromycin long-term. Fluclox = oral flucloxacillin long-term. Inh Coli = inhaled colistin (nebulised or inhaler). Inh Tob = inhaled tobramycin (nebulised or inhaler). RSI score = Reflux symptom index score, <13 normal. NA = not available.

**Table 2:** Demographic data for the non-CF patients.

Patient No	Age/yrs	Background disease	PPI yes/no*	Gastric juice pH
1	75	Oesophagitis	yes	2.4
2	56	Oesophagitis and Pyloroplasty	yes	6.6
3	65	Barrett’s Oesophagus and Hiatus Hernia	no	4.8
4	59	Hiatus Hernia	yes	2
5	45	Oesophagitis and Hiatus Hernia	n/a	1.4
6	42	Gastritis and Hiatus Hernia	yes	5.5
7	58	Oesophagitis and Hiatus Hernia	yes	4
8	80	Not known	n/a	4.7
9	50	Gastric ulcer	yes	8.4
10	78	Gastritis and Hiatus Hernia	n/a	1.6
11	73	Barrett’s Oesophagus	yes	5.1
12	55	Not known	n/a	5.2
13	68	Duodenal ulcer	n/a	6
14	65	Gastritis	n/a	1.7

All patients were off PPI or any other acid suppression medication 2 weeks before endoscopy procedure.
