# Supplementary material for: Microbiological profiles of sputum and gastric juice aspirates in Cystic Fibrosis patients
Source: Sci Rep. 2016 Jun 1;6:26985. doi: 10.1038/srep26985 (PMC4887896; doi:10.1038/srep26985)
Supplement: Supplementary Information [file srep26985-s1.pdf]

Microbiological profiles of sputum and gastric juice aspirates in Cystic Fibrosis patients

H Al-momani, A Perry, CJ Stewart, R Jones, A Krishnanan, AG Robertson, S Bourke, S Doe, SP Cummings, A Anderson, T Forrest, SM Griffin, M Brodlie, J Pearson, C Ward.

Online Data Supplement

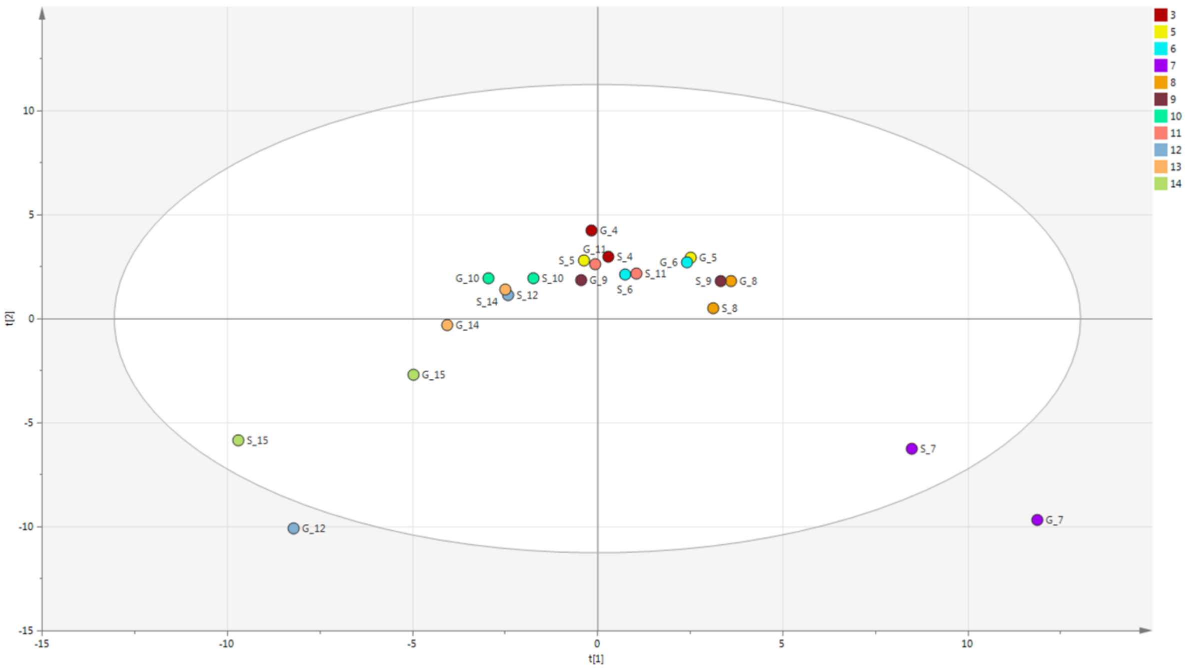

Figure E1: Partial least square decrement analysis (PLS-DA) score scatter plot. Each coloured circle represents a sample, where coloured grouping in based on an individual CF patient.

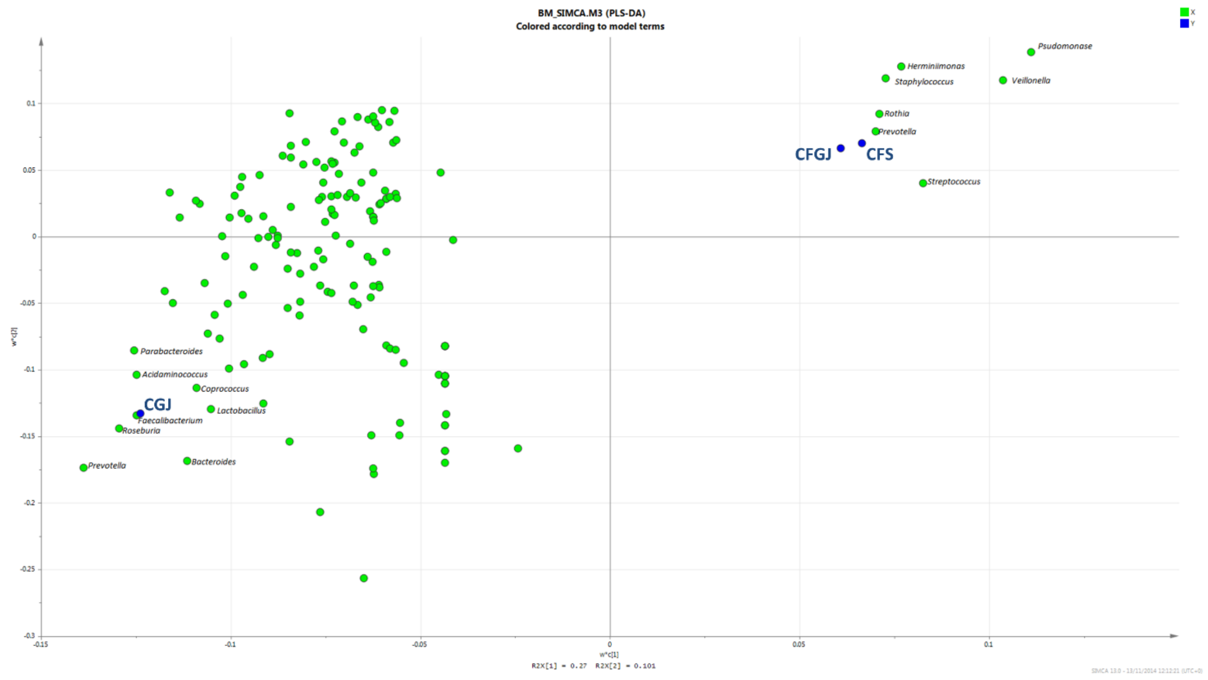

Figure E2: Partial least squares discriminant analysis (PLS-DA) loading plot. Non-CF gastric juice – GJ; CF gastric juice – CFGJ; Sputum samples – CFS. Some sample labels have been removed for ease of interpretation.

Table E1: CF gastric juice culture results

Gastric juice -1	*Candida albicans, Streptococcus mitis, Pseudomonas aeruginosa* and *Corynebacterium* spp
Gastric juice -2	*Candida kruzei and Aspergillus fumigatus*
Gastric juice -3	*Candida albicans and Staphylococcus hominis*
Gastric juice -4	*Candida* spp, *Streptococcus mitis, Staphylococcus haemolyticus, Neisseria spp, Brevundimonas* sp and *Delftia acidominus*
Gastric juice -5	*Candida albicans, Candida glabrata ,Candida kruzei, Pseudomonas aeruginosa , Achromobacter xylosoxidans, Lactobacillus fermentum* and A*lpha haemolytic streptococcus*
Gastric juice -6	*Candida albicans, Candida parasilosis, Candida kruzei* and *Streptococcus parasanguinis*
Gastric juice -7	*Candida glabrata* and *Candida albicans*
Gastric juice -8	*Candida albicans*
Gastric juice -9	*Candida glabrata, Candida albicans, Candida krusei* and *Lactobacillus* spp
Gastric juice -10	*Candida krusei, Acinetobacter* spp*, Lactobacilli gasseri* and *Micrococcus luteus*
Gastric juice -11	*Candida albicans, Candida parapsilosis , Candida glabrata* and *Staphylococcus hominis*
Gastric juice -12	*Candida albicans*, *Candida glabrata*, *Propionibacterium acnes* and *Pseudomonas aeruginosa*
Gastric juice -13	*Candida glabrata, Candida albicans* and *Candida krusei*
Gastric juice -14	*Candida albicans , Candida glabrata, Enterococcus faecium* and *Klebsiella pneumonia*
Gastric juice -15	*Candida lusitania , Candida parapsilosis, Pseudomonas aeruginosa* and *Lactobacillus gasseri*

Table E2: Sputum sample microbiology result

Sputum -1	*Candida albicans* , *Pseudomonas aeruginosa* and *Streptococcus mitis*
Sputum -2	*Streptococcus oralis, Achromobacter* spp and *Aspergillus fumigatus*
Cough swab -3	*Rothia mucilaginosa, Streptococcus mitis, Aspergillus fumigatus* and *Citrobacter koseri*
Sputum -4	*Candida albicans, Candida* spp, *Capnocytophaga sputigena, Staphylococcus aureus, Streptococcus mitis, Streptococcus parasanguinis, Aspergillus fumigatus, and Mycobacterium abscessus* subsp. *abscessus*
Sputum -5	*Candida albicans, Pseudomonas aeruginosa* and *Achromobacter xylosoxidans*
Sputum -6	*Candida albicans, Achromobacter xylosoxidans and Pseudomonas aeruginosa*
Sputum -7	*Rothia dentocariosa, Rothia mucilaginosa, Rothia aeria, Haemophilus parainfluenzae, Streptococcus mitis, Neisseria mucosa* and *Mycobacterium abscessus* subsp. *abscessus*
Cough swab -8	*Neisseria flavescens* , *Streptococcus salivarius, Actinomyces graevenitzii Pseudomonas aeruginosa, Rothia mucilaginosa and Stenotrophomonas maltophilia*
Sputum -9	*Pseudomonas aeruginosa* and *Aspergillus fumigatus*
Sputum -10	*Pseudomonas aeruginosa, Alpha haemolytic streptococcus* and *Burkholderia multivorans*
Sputum -11	*Candida albicans, Streptococcus mitis, Rothia mucilaginosa,* and *Pseudomonas aeruginosa*
Sputum -12	*Candida albicans, Pseudomonas aeruginosa, Streptococcus parasanquinis and Staphylococcus epidermidis*
Sputum -13	*Candida glabrata, Candida albicans, Candida parapsilosis, Pseudomonas aeruginosa, Staphylococcus aureus* and *Enterococcus faecium*
Sputum -14	*Candida parapsilosis, Staphylococcus aureus, Streptococcus salivarius, Neisseria* spp, *Streptococcus salivarius, Actinomyces odontolyticus, Pseudomonas aeruginosa, Haemophilus parainfluenzae* and *Exophiala dermatitidis*
Sputum -15	*Candida dubliniensis, Streptococcus gordonii* and *Pseudomonas aeruginosa*

Table E3 : Non-CF Gastric juice microbiology result

Gastric juice-1	No growth
Gastric juice -2	*Streptococcus angionosis, Alpha haemolytic streptococcus* and *Corynebacterium* spp
Gastric juice -3	*Serratia liquefaciens, Rahnella aquatilis and Neisseria* spp
Gastric juice -4	No growth
Gastric juice -5	No growth
Gastric juice -6	*Candida albicans, Coagulase negative staphyloccocus* and *Acinetobacter junii*
Gastric juice -7	No growth
Gastric juice -8	*Candida albicans, Alpha haemolytic streptococcus* and *Acinetobacter lwoffii*
Gastric juice -9	*Alpha haemolytic streptococcus*
Gastric juice -10	*Candida albicans*
Gastric juice -11	*Proteus mirabilus* and *klebsiella ozaenae*
Gastric juice -12	*Corynebacterium* spp and *Coagulase negative staphyloccous*
Gastric juice -13	*Pseudomonas aeruginosa* and *Alpha haemolytic streptococcus*
Gastric juice -14	No growth
